# The Cost of Metabolic Interactions in Symbioses between Insects and Bacteria with Reduced Genomes

**DOI:** 10.1128/mBio.01433-18

**Published:** 2018-09-25

**Authors:** Nana Y. D. Ankrah, Bessem Chouaia, Angela E. Douglas

**Affiliations:** a Department of Entomology and Genetics, Cornell University, Ithaca, New York, USA; b Department of Molecular Biology and Genetics, Cornell University, Ithaca, New York, USA; University of Miami; University of Hawaii at Manoa

**Keywords:** constraint-based modeling, flux balance analysis, nitrogen recycling, symbiosis, xylem-feeding insects

## Abstract

Various intracellular bacterial symbionts that provide their host with essential nutrients have much-reduced genomes, attributed largely to genomic decay and relaxed selection. To obtain quantitative estimates of the metabolic function of these bacteria, we reconstructed genome- and transcriptome-informed metabolic models of three xylem-feeding insects that bear two bacterial symbionts with complementary metabolic functions: a primary symbiont, Sulcia, that has codiversified with the insects, and a coprimary symbiont of distinct taxonomic origin and with different degrees of genome reduction in each insect species (Hodgkinia in a cicada, Baumannia in a sharpshooter, and Sodalis in a spittlebug). Our simulations reveal extensive bidirectional flux of multiple metabolites between each symbiont and the host, but near-complete metabolic segregation (i.e., near absence of metabolic cross-feeding) between the two symbionts, a likely mode of host control over symbiont metabolism. Genome reduction of the symbionts is associated with an increased number of host metabolic inputs to the symbiont and also reduced metabolic cost to the host. In particular, Sulcia and Hodgkinia with genomes of ≤0.3 Mb are calculated to recycle ∼30 to 80% of host-derived nitrogen to essential amino acids returned to the host, while Baumannia and Sodalis with genomes of ≥0.6 Mb recycle 10 to 15% of host nitrogen. We hypothesize that genome reduction of symbionts may be driven by selection for increased host control and reduced host costs, as well as by the stochastic process of genomic decay and relaxed selection.

## Abstract (precis)

Current understanding of many animal-microbial symbioses involving unculturable bacterial symbionts with much-reduced genomes derives almost entirely from nonquantitative inferences from genome data. To overcome this limitation, we reconstructed multipartner metabolic models that quantify both the metabolic fluxes within and between three xylem-feeding insects and their bacterial symbionts. This revealed near-complete metabolic segregation between cooccurring bacterial symbionts, despite extensive metabolite exchange between each symbiont and the host, suggestive of strict host controls over the metabolism of its symbionts. We extended the model analysis to investigate metabolic costs. The positive relationship between symbiont genome size and the metabolic cost incurred by the host points to fitness benefits to the host of bearing symbionts with small genomes. The multicompartment metabolic models developed here can be applied to other symbioses that are not readily tractable to experimental approaches.

## INTRODUCTION

The genome size of bacteria varies more than 50-fold from <0.2 to 12 Mb (1). This variation is largely representative of genetic capacity for function because the great majority of bacterial genomes are gene dense, with protein-coding regions accounting for 85 to 90% of the genome. Multiple factors influence bacterial genome size, including spatiotemporal variability in environmental conditions, nutrient availability, biotic interactions, and effective population size (1–4). Some of the bacteria with the tiniest genomes are intracellular bacterial symbionts in insects, and this trait is attributed largely to genomic decay arising from the vertical transmission of very small numbers of bacterial cells from the mother insect to her offspring (5, 6). Runaway genome reduction of these bacteria is countered by selection for metabolic function, specifically the synthesis of nutrients required by the insect host, and selection for reduced maintenance costs can also contribute to genome reduction (7). The most persuasive evidence for selection of small genome size comes from studies of free-living bacteria with large effective population size in low-nutrient environments (4, 8), but the possibility that small genome size may also be adaptive for insect endosymbionts has been raised (5, 9–11). Symbiont maintenance costs can be substantial because intracellular bacteria derive all their requirements from the surrounding host cell, consuming host nutrients that could otherwise have been utilized for host growth and reproduction. For example, the symbiont Buchnera in aphids is a major nutritional sink, estimated to consume 11 times more nitrogen than it provides to the insect host (12). However, the magnitude of these costs has never been quantified.

In this study, we investigated how the metabolic cost to the host of maintaining bacterial symbionts may vary with the genome size of the bacteria. We focused on xylem sap-feeding insects, which derive key nutrients (specifically, 10 essential amino acids and one or more B vitamins) from bacterial symbionts that are localized to specialized cells (bacteriocytes) and are transmitted vertically via the ovary of the female insect (13, 14). These associations are ideally suited to our purpose because, first, xylem sap is an extraordinarily nutrient-poor diet (15–17) exerting strong selection for metabolic efficiency in the insect symbiosis and, second, the genome size of the symbionts varies >10-fold, from 0.15 to 1.66 Mb in different xylem-feeding insects (5). Intriguingly, the nutritional function of these symbioses is partitioned between two bacteria, known as the primary symbiont and coprimary symbiont (18, 19). This condition is predicted to impose additional costs on the host, which has to support the nutritional requirements of two symbionts that mediate the same function as a single symbiont in other associations (11).

We studied three xylem-feeding insects: the spittlebug Philaenus spumarius, the sharpshooter Graphocephala coccinea, and the cicada Neotibicen canicularis. These insects possess the primary symbiont Sulcia muelleri (Bacteroidetes [henceforth referred to as Sulcia]), which produces 7 or 8 essential amino acids (20), and different coprimary symbionts that produce the complementary set of 3 or 2 essential amino acids and one or more B vitamins: Hodgkinia cicadicola (alphaproteobacterium [henceforth Hodgkinia]) in cicadas (21), Baumannia cicadellinicola (gammaproteobacterium [henceforth Baumannia]) in sharpshooters (14), and a bacterium allied to Sodalis glossinidius (gammaproteobacterium [henceforth Sodalis]) in spittlebugs of the tribe Philaeninae (22). The origin of these associations has been dated provisionally to 260 to 280 million years ago (mya) for Sulcia (20), ∼190 mya for Hodgkinia in cicadas (21), ∼80 mya for Baumannia in sharpshooters (23), and more recently for Sodalis in philaenine spittlebugs (22).

We hypothesized that the cost to the insect host of maintaining the bacteria may be reduced by metabolic efficiencies of the symbioses, including limited overlap between the metabolic outputs from the primary and coprimary symbionts and efficient bacterial recycling of host-derived nitrogenous compounds to essential amino acids returned to the host, and that these metabolic traits would be particularly evident in symbioses with more ancient coprimary symbionts with very small genomes. To test these predictions, we applied metabolic modeling techniques, which provide quantitative predictions of metabolic flux within individual partners, as well as between the bacteria and the insect host (12, 24–26). For each symbiosis, we reconstructed genome-scale metabolic models for each symbiont, together with a transcriptome-informed model for the insect bacteriocyte, and then combined these individual models to generate a three-compartment model with flux between the partners. Quantitative flux estimates were inferred by flux balance analysis (FBA), which optimizes flux to a desired outcome (objective function) (27) and flux variability analysis (FVA), which determines the range of fluxes that each reaction can achieve while maintaining the optimized objective function (28). Importantly, interpretation of the metabolic comparisons across the different bacterial symbionts is not confounded by phylogenetic differences between the bacteria because the metabolic reactions used in our models are generic to all the bacterial taxa under study. Our analyses confirmed our prediction of very little overlap in outputs between the bacterial symbionts in all symbioses and revealed reduced metabolic costs of the symbioses with more ancient coprimary symbionts.

## RESULTS

### The metabolic networks of the symbiotic bacteria and their hosts.

**FIG 1:**
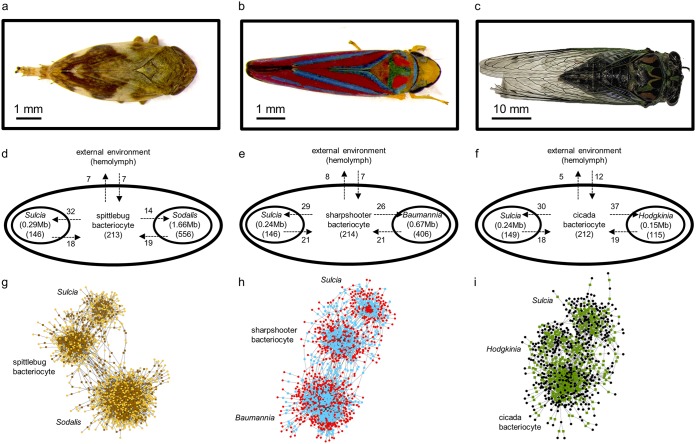
Metabolic interactions in xylem-feeding insect-bacterial symbiosis. (a to c) The insects used in this study (a) spittlebug (Philaenus spumarius), (b) sharpshooter (Graphocephala coccinea), and (c) cicada (Neotibicen canicularis). (d to F) Model structure showing species compartments and metabolites exchanged between each compartment for (d) spittlebug, (e) sharpshooter, and (f) cicada symbiosis. Bacterial genome size and the total number of metabolites in each compartment are shown in parentheses. The number of input and output metabolites for each compartment is displayed alongside the arrows. (g and h) Metabolic network maps of integrated three-partner (g) spittlebug, (h) sharpshooter, and (i) cicada models. The prefuse force-directed algorithm was used for generating the network layout and visualized with Cytoscape_v3.4.0. Circles (gold, red, and black) represent metabolites, and squares (brown, blue, and green) represent reactions.

We reconstructed the metabolic network of the primary symbiont (Sulcia), the coprimary symbiont, and the insect host for the three xylem-feeding insects (Fig. 1a to c). Consistent with their small genomes, the bacterial symbionts possess fewer metabolism-related genes that support fewer reactions and metabolites than free-living bacteria such as Escherichia coli (Table 1; Fig. 1d to f). The metabolic capabilities of the primary symbiont Sulcia are highly conserved across the three insects (Table 1), with a core set of 81 intracellular metabolic reactions contributing 89 to 94% of the total reactions in each Sulcia network (see Fig. S1 and Table S1a in the supplemental material). The coprimary symbionts vary in their metabolic capabilities, with the 50 reactions in Hodgkinia (in the cicada) representing just 14% of the 370 reactions in Baumannia (in the sharpshooter) and 9% of the 578 reactions in Sodalis (in the spittlebug) (Table 1). Overall, the three coprimary symbionts have a shared set of just 27 reactions, constituting 5 to 54% of the total reactions in each symbiont (Fig. 1d to f; Fig. S1 and Table S1A). The metabolic networks of the insect hosts each comprise 213 intracellular reactions and 212 to 214 metabolites (Table 1 and Fig. 1d to f).

**TABLE 1:** The bacterial and insect metabolic models used in this study

Symbiosis	No. of genes	No. of reactions	No. of unique metabolites
Spittlebug			
Sulcia	82	86	146
Sodalis	400	578	556
Philaenus spumarius	279	213	213
Integrated model	761	877	598
Sharpshooter			
Sulcia	74	88	146
Baumannia	234	370	405
Graphocephala coccinea	321	213	214
Integrated model	629	671	484
Cicada			
Sulcia	83	91	149
Hodgkinia	37	50	115
Neotibicen canicularis	413	213	212
Integrated model	533	354	365
E. coli K-12^*a*^			
MG1655	1,366	2,251	1,136

a Data from reference 27.

10.1128/mBio.01433-18.2 FIG S1 Overview of reactions and metabolites for xylem feeder bacterial symbionts. Reactions and metabolites are colored red (shared between all three primary or companion bacterial partners), blue (shared between any two primary or companion bacterial partners), and green (unique to a single bacterium). Download FIG S1, PDF file, 0.02 MB. Copyright © 2018 Ankrah et al. 2018 Ankrah et al. This content is distributed under the terms of the Creative Commons Attribution 4.0 International license.

10.1128/mBio.01433-18.5 TABLE S1 (a) Comparison of reaction and metabolite content of bacterial genome scale metabolic models. (b) Flux variability analysis for spittlebug symbiosis. (c) Flux variability analysis for sharpshooter symbiosis. (d) Flux variability analysis for cicada symbiosis. (e) List of inputs and outputs and their predicted fluxes for bacterial partners (Sulcia and Sodalis) in three-compartment spittlebug model. (f) List of inputs and outputs and their predicted fluxes for bacterial partners (Sulcia and Baumannia) in three-compartment sharpshooter model. (g) List of inputs and outputs and their predicted fluxes for bacterial partners (Sulcia and Hodgkinia) in three-compartment cicada model. (h) Metabolites shared between bacterial partners (part i) and unique metabolites transported by individual bacterial partners (part ii). (i) Nitrogen utilization by bacterial symbionts. Download Table S1, XLSX file, 0.24 MB. Copyright © 2018 Ankrah et al. 2018 Ankrah et al. This content is distributed under the terms of the Creative Commons Attribution 4.0 International license.

For each symbiosis, the metabolic networks of the bacterial symbionts and host were combined via transport reactions to form an integrated three-compartment model (Fig. 1g to i). We used these three-compartment metabolic models to determine the metabolite flux between the partners in each symbiosis. Specifically, we quantified the metabolite outputs from the bacterial symbionts to the host and other bacterial symbionts and the metabolite inputs from the host to the bacterial symbionts by FBA. To assess whether the flux through reactions mediating interactions between host and symbionts are tightly constrained, the minimal and maximal fluxes through each reaction were determined by FVA, while maintaining a fixed maximal theoretical growth yield of the bacterium. The range of fluxes for ∼88% of all reactions in all three models varied by less than 1 mmol g dry weight^−1^ h^−1^ (see Table S1b to d and Fig. S2 in the supplemental material), and 55 to 80% of the transport reactions between host and symbiont varied in flux by less than 1% (Table S1b to d and Fig. S3 in the supplemental material). Due to the low flux variability in our models, all fluxes reported in the rest of this article are optimal fluxes predicted by FBA.

10.1128/mBio.01433-18.3 FIG S2 Variability in metabolic flux predictions in spittlebug, sharpshooter, and cicada symbioses analyzed by flux variability analysis (FVA). Flux ranges are calculated as the difference between the maximum and minimum flux distributions resulting in the same growth objective value. Download FIG S2, PDF file, 0.01 MB. Copyright © 2018 Ankrah et al. 2018 Ankrah et al. This content is distributed under the terms of the Creative Commons Attribution 4.0 International license.

10.1128/mBio.01433-18.4 FIG S3 Variation in symbiont transport flux relative to optimal (maximum) flux in spittlebug, sharpshooter, and cicada symbioses. Variation around maximum is calculated by dividing the range of flux variation (obtained by FVA) by the optimal flux (calculated by FBA) and is expressed as a percentage. Download FIG S3, PDF file, 0.01 MB. Copyright © 2018 Ankrah et al. 2018 Ankrah et al. This content is distributed under the terms of the Creative Commons Attribution 4.0 International license.

### Metabolic outputs from the symbionts.

**FIG 2:**
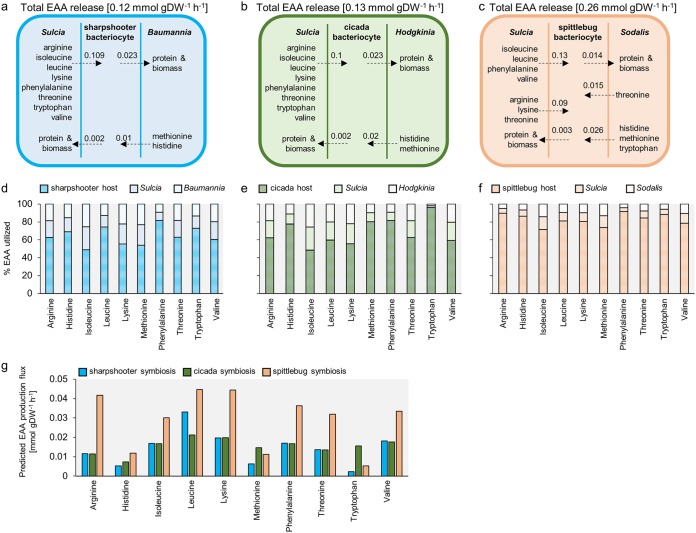
Comparison of EAA synthesis fluxes and utilization profiles for three-compartment insect-bacterial symbioses. (a to c) *In silico* predictions of EAA export by bacteria in sharpshooter, cicada, and spittlebug symbiosis. (d to f) Comparison of EAA utilization profiles for bacteria and host in (d) sharpshooter, (e) cicada, and (f) spittlebug symbiosis. (g) *In silico* predictions of EAA production in sharpshooter, cicada, and spittlebug symbiosis.

Our first analysis focused on the principal metabolic function of the symbiotic bacteria, the production of essential amino acids (EAAs). The metabolic models supported the net release of every EAA synthesized by each bacterium in the sharpshooter and the cicada symbioses: 8 EAAs by Sulcia and the two remaining EAAs (histidine and methionine) by the coprimary symbiont (Fig. 2a and b). The metabolic model for the spittlebug symbiosis also supported the net release of the 7 EAAs synthesized by Sulcia and 4 of the 6 EAAs synthesized by the coprimary symbiont Sodalis, comprising histidine, methionine, and tryptophan, which are not synthesized by Sulcia in this symbiosis, and threonine, which was also produced by Sulcia (Fig. 2c). In our models, the EAAs arginine and lysine synthesized by Sodalis were not released.

In our simulations, the total flux of EAA release was 0.26 mmol g dry weight^−1^ h^−1^ for the spittlebug symbiosis, 0.12 mmol g dry weight^−1^ h^−1^ for the sharpshooter, and 0.13 mmol g dry weight^−1^ h^−1^ for the cicada symbioses (Fig. 2a to c; Table S1e to g). The host was the largest sink for all EAAs derived from the symbionts, consuming between 49 and 96% of every EAA produced (Fig. 2d to f). The fluxes of EAA release varied by an order of magnitude across the different EAAs, with leucine and lysine consistently released at high fluxes (Fig. 2g). All the EAAs derived from coprimary symbionts had low release fluxes (Fig. 2a to c). Histidine and methionine release from Baumannia represented just 8% of the total EAAs released in the sharpshooter symbiosis and the equivalent value for Hodgkinia in the cicada was 15%. Sodalis contributed 16% of the total EAAs released in the spittlebug symbiosis, comprising methionine and histidine (8%), tryptophan (2%), and threonine (6%).

**FIG 3:**
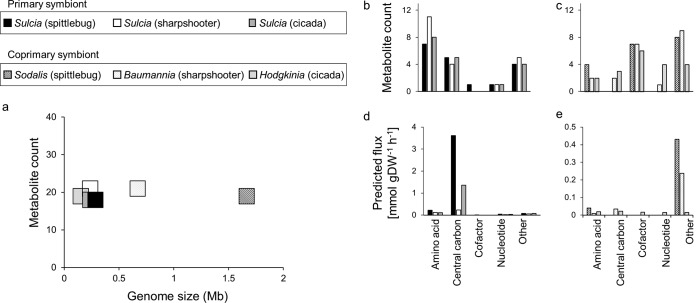
Comparison of metabolites exported by bacteria from three-compartment insect-bacterial symbioses based on metabolite counts and metabolite fluxes. (a) Relationship between bacterial genome size and number of metabolic outputs exported to the host. (b to e) Metabolic outputs to bacterial compartments based on (b and c) metabolite counts and (d and e) metabolite fluxes. (Note the difference in scales of flux between the primary symbionts [left] and coprimary symbionts [right].) Fluxes of individual metabolite production and consumption are provided in Table S1e to g.

Our models also revealed that the symbionts release a range of metabolites in addition to EAAs. The total number of metabolites released was 18 to 21, independent of genome size (Fig. 3a to c). However, the flux of metabolites exported from primary symbionts was higher than that from coprimary symbionts (Fig. 3d and e), and the differences were 2-fold for the sharpshooter symbiosis, 8-fold for the spittlebug symbiosis, and 18-fold for the cicada symbiosis (Table S1e to g).

Sulcia in all three symbioses released the same set of five central carbon metabolites: succinate, fumarate, xylulose-5-P, glycerate-1,3-P, and dihydroxyacetone P (Table S1e to g). The compounds released from the coprimary symbionts varied between the different symbioses (Fig. 3c and e and Table S1e to g). In particular, ammonia constituted the highest flux of material released from Sodalis (0.15 mmol g dry weight^−1^ h^−1^) and Hodgkinia (0.015 mmol g dry weight^−1^ h^−1^), while acetate accounted for the highest flux of material released from Baumannia (0.23 mmol g dry weight^−1^ h^−1^) (Table S1e to g). In our models, the ammonia was metabolized by the host to glutamine, and glutamine to glutamate, via host-encoded glutamine synthetase and glutamate synthase, respectively, and the acetate was assimilated into the host’s central carbon metabolism.

The overlap in outputs from the primary and coprimary symbionts in each association comprised up to two metabolites: ammonia and threonine in the spittlebug, acetate in the sharpshooter, and acetate and AMP in the cicada (Table S1h, part i). For each association, 16 to 20 unique metabolites were released from the primary and coprimary symbionts (Table S1H, part ii).

**FIG 4:**
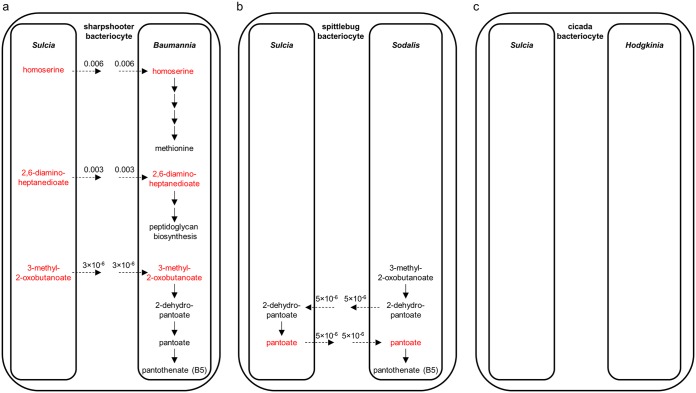
Metabolite cross-feeding between bacterial partners. Shown are metabolites exchanged exclusively between bacterial partners in (a) spittlebug, (b) sharpshooter, and (c) cicada symbiosis. Metabolites produced by Sulcia are colored red. Inferred fluxes for metabolite groups assimilated and released by bacteria are given in mmol g dry weight^−1^ h^−1^.

We also investigated the incidence of cross-feeding of metabolites synthesized by one symbiont and required exclusively by the other symbiont (and not the host). Five cross-fed metabolites were identified, each unique to a single symbiosis (Fig. 4). The sharpshooter symbiosis had three instances of transfer from the primary symbiont Sulcia to the coprimary symbiont Baumannia, one contributing to Baumannia peptidoglycan synthesis and two to products Baumannia delivered to the host (homoserine, a precursor of the EAA methionine, and 3-methyl-2-oxobutanoate, a precursor of the B vitamin pantothenate) (Fig. 4). The two exchanged metabolites in the spittlebug symbiosis are intermediates in the synthesis of the B vitamin pantothenate (Fig. 4). The cicada symbiosis has no metabolites that are transferred exclusively between symbionts (Fig. 4).

### Metabolic inputs to the symbionts from the host.

**FIG 5:**
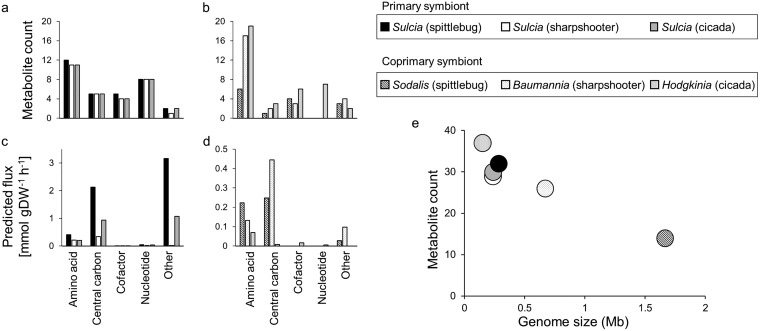
Comparison of metabolites consumed by bacteria from three-compartment insect-bacterial symbioses based on metabolite counts and metabolite fluxes. Shown are metabolic inputs to bacterial compartments based on (a and b) metabolite counts and (c and d) metabolite fluxes. (Note the difference in scales of flux between the primary symbionts [left] and coprimary symbionts [right].) (e) Relationship between bacterial genome size and number of metabolic inputs derived from the host. Fluxes of individual metabolite production and consumption are provided in Table S1e to g.

In terms of metabolite counts, the principal metabolites imported by both primary and coprimary symbionts were amino acids and their derivatives (Fig. 5a and b), and in quantitative terms, central carbon intermediates were dominant (Fig. 5c and d). For Sulcia, the amino acid with the highest import flux was glutamate (utilized in reactions in EAA synthesis), while fructose 6-phosphate and malate were the chief central carbon imports (Table S1e to g). For the coprimary symbionts, the dominant inputs varied with species. The chief nitrogen and carbon inputs, respectively, were glutamine and 6-phospho-d-glucono-1,5-lactone for Sodalis, serine and fructose for Baumannia, and ribose-5-P and cystathionine for Hodgkinia (Table S1e to g).

The number of metabolic inputs to the bacterial symbionts varied inversely with bacterial genome size (Fig. 5e), ranging from 14 metabolic inputs to Sodalis (1.66 Mb genome) to 37 inputs to the bacterium with the smallest genome, Hodgkinia (0.15 Mb genome). In parallel, the number of host-derived metabolites shared between the primary and coprimary symbionts increased with reduced genome size of the coprimary symbiont, from two shared metabolites for the spittlebug symbiosis, through 8 for the sharpshooter, to 15 for the cicada symbiosis. The two shared metabolites in the spittlebug symbiosis, glutamine and tyrosine, were also shared between the primary and coprimary symbionts in the other symbioses. (Table S1h, part i). For each association, the primary and coprimary symbionts imported 12 to 30 unique metabolites from the host (Table S1h, part ii).

Taken together, these analyses reveal that, as the metabolic scope of the bacterial symbionts declines with genome reduction, the number of host metabolites required to support bacterial metabolism increases. This relationship is accompanied by an increased overlap in the number of host-derived metabolites utilized by the primary and coprimary symbionts.

### The metabolic cost of the symbiosis to the host.

**FIG 6:**
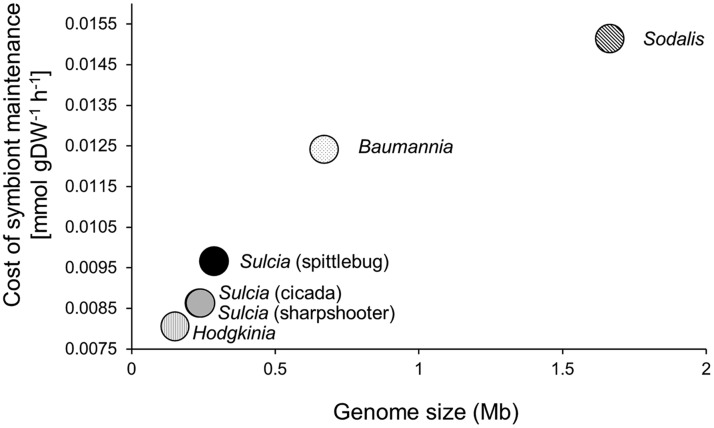
Bacterial maintenance costs incurred by host insects. Bacterial maintenance costs are inferred from reductions in growth flux the host incurs by harboring a bacterium.

To estimate the cost of maintaining bacterial symbionts by each host, simulations were performed comparing host growth yields in the presence and absence of biomass production by either the primary or coprimary symbiont. For these simulations, the uptake fluxes for the main sources of C, N, P, and S (glucose, fructose, ammonium, phosphate, and sulfate) were capped at the observed uptake fluxes in the three-compartment model by setting the lower bounds of the uptake reactions to the predicted uptake fluxes with both symbionts present. Our simulations indicated that the cost of maintaining bacterial partners by the host decreased with declining bacterial genome size (Fig. 6).

**FIG 7:**
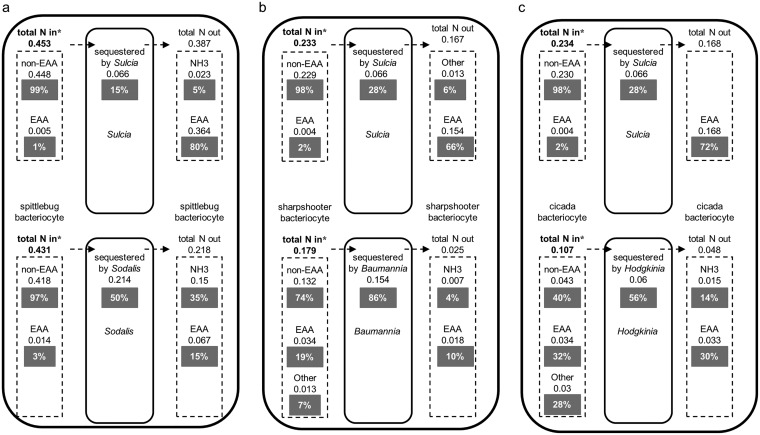
Nitrogen utilization by bacterial symbionts. Inferred fluxes for total nitrogen assimilated and released by bacteria are calculated by multiplying the flux through a metabolite transport reaction by the N stoichiometry of the given metabolite. (a) Spittlebug. (b) Sharpshooter. (c) Cicada. Broken arrows represent transport fluxes between host and symbionts. Reaction fluxes (mmol g dry weight^−1^ h^−1^) are shown below each metabolite transport class. Percentages represent the proportion of flux through each metabolite transport class (e.g., non-EAA transport input flux) relative to the total N input transport flux into each symbiotic partner (denoted by bold text with an asterisk). Individual metabolite fluxes are shown in Table S1i.

We extended the analysis of metabolic costs to quantify the supply of host-derived N to EAA production, the key metabolic function of the symbionts. For Sulcia, EAA output was equivalent to 66 to 80% of host-derived N (Fig. 7a to c; Table S1i). The coprimary symbionts were less efficient in their transformation of host N into EAAs delivered back to the host, at 30% for Hodgkinia (Fig. 7c), 15% for Sodalis (Fig. 7a), and 10% for Baumannia (Fig. 7b).

## DISCUSSION

Metabolic modeling is widely used in biotechnological applications to predict and explain the metabolic consequences of specific genetic manipulations of metabolism-related genes, such as gene deletions and altered gene expression (29–31), and it is also increasingly being applied to investigate metabolic interactions, especially among microorganisms (24, 32–36). These modeling studies provide a powerful route to identify feasible metabolic solutions and to generate quantitative hypotheses for empirical testing, recognizing that the model outputs are not intended to be a perfect representation of the biological system under study. The constraint-based modeling approach adopted here generated optimized metabolite flux distributions across three linked metabolic networks (two bacterial symbionts and their host), and they successfully captured the core function of the symbiotic bacteria, comprising their synthesis and release of EAAs to the host (Fig. 2). More broadly, the models yield predictions of the flux of metabolites transferred between the partners that cannot be obtained from enumeration of the metabolism gene content of bacterial symbionts.

Consistent with computational analyses of bacteria in other habitats (37), the number of metabolic inputs to the symbionts varies inversely with symbiont genome size (Fig. 5e). In other words, the bacterial symbionts with small genomes consume a greater diversity of host metabolites than bacteria with larger genomes in xylem-feeding insects. An important process contributing to genome reduction in the bacterial symbionts is genomic decay (see the introduction), which is predicted to lead to a generalized decline in the integrity of the metabolic network of bacteria, but this is unlikely to be a complete explanation for our observation because the number of metabolites released from the bacteria does not vary with genome size (Fig. 3a). We hypothesize that it may be advantageous to the host for its symbionts to require multiple metabolic inputs. Specifically, some metabolic inputs may be points of host control over symbiont metabolism, as has been demonstrated for the Buchnera symbiont in aphids (38), and the regulated supply of multiple metabolites may provide for more robust and precise host controls over symbiont growth and function. Thus, among the three symbioses investigated in this study, we predict that host control over EAA release and growth yields of the coprimary symbiont is greater for the cicada association with Hodgkinia (37 inputs) than for the sharpshooter association with Baumannia (26 inputs) and the spittlebug association with Sodalis (14 inputs). These metabolic controls may operate in conjunction with both controls over transport across the host-symbiont interface and also host effector molecules, including immune-related products, to regulate symbiont growth yields and nutrient release fluxes. Such mechanisms have not, to date, been investigated in xylem-feeding insect symbioses, but they have been identified in other intracellular symbioses. For example, an amino acid transporter expressed in the aphid bacteriocyte has been functionally characterized in the aphid symbiosis (39), the antimicrobial peptide coleoptericin A has been implicated in the regulation of symbiont proliferation in Sitophilus weevils (40), and cysteine-rich peptides, which promote nutrient release from bacterial symbionts in plant roots (41, 42), have been identified in some insect symbioses (43, 44).

Implicit in the hypothesis that symbiont metabolic function and growth are regulated by metabolic inputs from the host is that the supply of these inputs can limit metabolic flux and biomass production in the symbiont. Where a single host-derived metabolite is an input for both the primary and coprimary symbionts, between-symbiont competition can ensue with deleterious consequences, including increased allocation of symbiont resources to competitive traits instead of services to the host and reduced fitness of both symbionts and host (45–48). Our models suggest that between-symbiont competition could be particularly intense because proportionately more shared metabolites than inputs to single symbionts are allocated to biomass production, rather than to EAA release to the host.

How might competition for host-derived metabolites that are shared between the primary and coprimary symbionts be constrained? Two complementary processes may be involved. First, the host may provide an excess of shared metabolites but limit the supply of nutrients that are exclusive to each symbiont. Symbiont growth and EAA production could, thereby, be controlled by the exclusive inputs, preventing overconsumption of the shared metabolites. Additionally or alternatively, competitive interactions may be suppressed by host-mediated segregation of the symbionts. Specifically, symbiont access to host metabolites is constrained by a host membrane, the symbiosomal membrane, which bounds each bacterial cell and, where investigated, has highly selective transport properties exerting substantial controls over host metabolite supply to the symbionts (49, 50). Metabolic segregation is not, however, complete because limited cross-feeding of metabolites between the two symbionts was identified in the models for two of the three associations (in the spittlebug and sharpshooter). Interestingly, a majority (4 out of 5) of the cross-fed metabolites contribute to the synthesis of EAAs and B vitamins that are released to the host (Fig. 4). This pattern raises the possibility that selection may favor metabolic interactions between the primary and coprimary symbionts that contribute directly to host nutrition.

The second robust pattern that emerged from our analysis was that symbionts with a smaller genome are less costly to the host than symbionts with a larger genome (Fig. 6). The underlying reason is that symbionts with highly reduced genomes have very small metabolic networks that are dominated by linear pathways, with few metabolic reactions that shunt host-supplied precursors away from EAA synthesis to other biochemical pathways. These results suggest that selection for metabolic efficiency may favor genome reduction in these bacteria.

We predicted that selection to minimize metabolic costs of the symbiosis would be especially high in symbioses subsisting on xylem sap, which (as considered in the introduction) is very nutrient poor, especially in organic carbon and nitrogen. With respect to carbon, the coprimary symbiont Hodgkinia imposes minimal costs because it has no capacity for independent energy production (21) (Table S1d), but the demand for central carbon compounds by the other symbionts is substantial, accounting for 37 to 66% of their total inputs (Table S1e to g). The host is expected to maintain tight metabolic controls over the supply of these major compounds. Consistent with our argument (above) that the host preferentially limits the flux of metabolites unique to each symbiont rather than shared metabolites, the major organic carbon inputs differ between the primary and coprimary symbionts in each symbiosis. Turning to nitrogen, all the symbionts impose a net demand on host nitrogen resources, but the magnitude of the cost varies widely among the different symbionts. The efficiency of Sulcia nitrogen metabolism, with up to 80% of input N released back to the host as EAAs, greatly exceeds the calculated value of 60% for the intracellular symbiont Portiera in the whitefly symbiosis (24) and the 9% estimated for the Buchnera symbiont in aphids (12). We recognize, however, that the inclusion of metabolite transport reactions that are not energetically costly to the symbionts or host may potentially underestimate the costs associated with symbiont maintenance and may affect the symbiont cost estimations. The coprimary symbionts are appreciably less efficient (10 to 30%) than Sulcia, and the difference can be attributed to the low-output flux of EAAs synthesized by the coprimary symbionts and the high-output flux of ammonia, especially from Sodalis.

We conclude by considering the contribution that symbioses in xylem-feeding insects can make to our general understanding of metabolic function in symbiotic microorganisms. Previous research based on analysis of the gene content of the symbionts has revealed how selection pressures exerted in the symbiosis have led to the remarkable evolutionary convergence of phylogenetically diverse coprimary symbionts to produce EAAs that precisely complement the EAA biosynthetic function of the primary symbiont Sulcia (14, 19). The genome-scale modeling described here provides quantitative validation of these conclusions and demonstrates that the metabolic cost to the host of maintaining intracellular symbionts declines with decreasing genome size of the symbiont, despite a parallel increase in the number of host-derived metabolites required by the symbiont. These results provide a quantitative basis for the argument that genome reduction of symbionts, especially in hosts utilizing grossly nutrient-poor diets such as xylem sap, may not be driven entirely by genetic drift and relaxed selection (see the introduction), but may be of selective advantage to the host. The generality of the relationships between symbiont genome size and metabolic traits identified in these xylem-feeding insects can be investigated using phylogenetically different symbionts and hosts on diets of different nutritional profiles.

## MATERIALS AND METHODS

### The insects.

Adults of Philaenus spumarius (Linneus, 1758), informally known as the meadow spittlebug, and Graphocephala coccinea (Forster, 1771), a sharpshooter informally known as the red-banded leafhopper, were collected from vegetation surrounding Beebe Lake, Ithaca, NY, in June 2014 and July 2015, respectively. Mature nymphs of the dog-day cicada Neotibicen canicularis (Harris, 1841) were collected from tree trunks at Lansing, Ithaca, NY, and retained in the laboratory for up to 3 days after they had molted to adulthood. Species identification was carried out using taxonomic keys (51–53) (voucher specimen CU1268 held in the Cornell University Insect Collection). For bacterial genome sequencing, bacteriomes were dissected from each insect in ice-cold filter-sterilized phosphate-buffered saline (PBS) and transferred to 70% ethanol. Total DNA was extracted using the DNeasy blood and tissue kit (Qiagen) “tissue extraction” protocol and eluted in 50 µl AE buffer (Qiagen). For transcriptome analysis, replicate samples of whole bodies and freshly dissected bacteriomes of each species (two samples for N. canicularis, four for G. coccinea, and six for P. spumarius) were transferred to RNAlater (ThermoFisher) and RNA was extracted with the RNeasy kit (Qiagen) “tissues” protocol, including treatment with RNase-free DNase I (Qiagen) for 15 min at room temperature, following the manufacturer’s instructions. The final product was eluted in 50 µl RNase-free water.

### DNA library preparation and sequencing of bacterial genomes.

The extracted DNA (1 to 2 µg per sample) was fragmented using an S2 ultrasonicator (Covaris) to obtain 700-bp fragments, which were end repaired with the End repair mix LC (Enzymatics) and A-tailed with the Klenow 3′→5′ exo-enzyme (Enzymatics). Universal Y-shaped adaptors were ligated using A-T ligation, adaptor-ligated DNA was purified and size-selected using AMPure XP beads (Agencourt), and DNA was subjected to 14 cycles of PCR amplification with barcoded Illumina index primers (see Table S2 in the supplemental material). The amplified DNA was purified with AMPure XP beads and eluted in 15 µl buffer EB (Qiagen). Concentrations were determined by Qubit 2.0 fluorometer (Thermo Fisher) with the DNA HS assay, yielding 5.8 to 14.1 ng DNA µl^−1^. Library quality was assessed on a Bioanalyzer, and equimolar pools were subjected to 2× 150-bp paired-end sequencing on an Illumina HiSeq2500 platform.

10.1128/mBio.01433-18.6 TABLE S2 TrueSeq index primers. Download Table S2, XLSX file, 0.01 MB. Copyright © 2018 Ankrah et al. 2018 Ankrah et al. This content is distributed under the terms of the Creative Commons Attribution 4.0 International license.

Following the removal of adaptors and quality filtering, the DNA reads were used to assemble the genome of each bacterium. First, the total bacteriome metagenome was assembled using the CLC genomics workbench (version 3.6 CLC, Inc., Aarhus, Denmark). BLASTn (BLAST version 2.2 [54]) searches of the resulting contigs were performed against *ad hoc*-built databases created using the publicly available genomes of each bacterium (see Table S3 in the supplemental material), and the reads associated with the contigs for each bacterium were extracted separately and reassembled using SPAdes version 3.5 (55) to generate the bacterial genomes. Genome annotations were carried out on RAST (56), using Glimmer 3 as an open reading frame (ORF) caller for all bacteria, except Hodgkinia, which uses an alternative genetic code (21). The contigs from our Hodgkinia genome assembly were used to perform a BLASTn search against a reference Hodgkinia genome (PRJNA246493 [57]). For this search, a gene was considered to present when the BLASTn search results matched a single entry in the reference Hodgkinia genome and matched the total length of our contigs.

10.1128/mBio.01433-18.7 TABLE S3 List of reference genomes. Download Table S3, XLSX file, 0.01 MB. Copyright © 2018 Ankrah et al. 2018 Ankrah et al. This content is distributed under the terms of the Creative Commons Attribution 4.0 International license.

### Illumina RNA-seq library preparation.

Transcriptome sequencing (RNA-seq) libraries were generated from 2 µg total RNA per replicate, using a published protocol (58) with minor modifications. Poly(A)^+^ RNA was purified using Dynabeads oligo(dT) (Life Technologies) according to the manufacturer’s protocol and fragmented by incubation at 94°C for 2 min to generate long fragments (>700 bp). cDNA was synthesized using Superscript II reverse transcriptase (Invitrogen) following the manufacturer’s protocol, and the resulting cDNA was purified using RNA Clean XP magnetic beads (Agencourt). Strand-specific libraries were generated with dUTP for second-strand synthesis. Double-stranded cDNA was end repaired, A-tailed, and ligated to adaptors as for the DNA library preparation (described above), and the resultant cDNA was purified and size selected to obtain 750-bp fragments. The uracil-containing second strand was then digested using uracil DNA glycosylase (Enzymatics), and cDNA was subjected to 15 cycles of PCR amplification using barcoded Illumina index primers (Table S2). The final cDNA was purified using AMPure XP beads (Agencourt) and eluted in 15 µl buffer EB (Qiagen). The concentration was determined by Qubit (as described above), yielding 6.33 to 37.5 ng RNA µl^−1^, library quality was checked by Bioanalyzer, and equimolar pools were used for 150-bp paired-end sequencing on an Illumina HiSeq2500.

### RNA-seq expression analysis.

The raw reads were trimmed to remove adaptors and quality filtered, retaining reads with an average quality score of >30. The reads were mapped against the reference genomes of the bacteria obtained in this project (Table S3), and the mapped reads were excluded from the data set. High-quality reads from each bacteriome and body sample were then assembled individually using Trinity version 2.1.1 (59) with default settings. Transcripts from bacteriome and body samples were then merged using CD-HIT version 4.6.6 (60), considering a similarity threshold of 90%. ORF detection was carried out using the Transdecoder suite version 2.0.1 (https://transdecoder.github.io/) with default settings. The transcriptome was annotated using the Trinotate pipeline version 2.0.1 (https://trinotate.github.io/) and local BLAST (54) against SwissProt with an E value cutoff of 1e−5. The completeness of the transcriptomes was assessed with BUSCO v3 (61): our transcriptomes included 75 to 87% of the 1,658 single-copy orthologous insect genes in OrthoDB v9 (62) (see Table S4 in the supplemental material). Expression analysis was conducted with Trinity utility suite (https://github.com/trinityrnaseq/trinityrnaseq/). The reads from each sample were aligned against the reference transcriptome using the align_and_estimate_abundance.pl script with bowtie2 as the aligner and RSEM (63) as the abundance estimation method to determine transcripts per million mapped reads (TPM). The expression level of the different transcripts was then normalized to the expression of the lowest transcript. Specifically, the mean TPM for each gene was divided by the lowest nonzero count and rounded to the nearest integer. Transcripts with the lowest nonzero TPM received a normalized expression level of 1, and all other transcripts received multiples of 1. Transcripts with zero TPM counts (i.e., very-low-abundance transcripts with lengths less than the mean fragment length [63]) were assigned the lowest TPM values in each replicate and normalized as described above. Zero-TPM transcripts were used only for calculating the total protein content for each insect host.

10.1128/mBio.01433-18.8 TABLE S4 Completeness of host transcriptomes, as analyzed by BUSCO. Download Table S4, XLSX file, 0.01 MB. Copyright © 2018 Ankrah et al. 2018 Ankrah et al. This content is distributed under the terms of the Creative Commons Attribution 4.0 International license.

### Metabolic model reconstruction and analysis.

Genome-scale metabolic models were generated for the symbiotic bacteria (Sulcia [iNA82] and Sodalis [iNA400] from the spittlebug, Sulcia [iNA74] and Baumannia [iNA234] from the sharpshooter, and Sulcia [iNA83] and Hodgkinia [iNA37] from the cicada) (see Table S5a to f in the supplemental material) following the procedure in reference 24, as described in Text S1 in the supplemental material. For the host models, reactions capable of generating or consuming dead-end metabolites in each bacterial model were identified and incorporated in the draft reconstruction where the cognate metabolism genes were detected in the host transcriptome (Table S5g to i). Orphan reactions (non-gene-associated reactions) (Table S5j) were added to fill gaps in all the metabolic networks. All metabolic networks were visualized using Cytoscape_v3.4.0 (64), and model testing was conducted in COBRA Toolbox version 3.0 (65) run in Matlab (The MathWorks, Inc., Natick, MA), using the Gurobi solver (66).

10.1128/mBio.01433-18.1 TEXT S1 Supplementary methods. Download Text S1, DOCX file, 0.02 MB. Copyright © 2018 Ankrah et al. 2018 Ankrah et al. This content is distributed under the terms of the Creative Commons Attribution 4.0 International license.

10.1128/mBio.01433-18.9 TABLE S5 (a) Stand-alone Sulcia (spittlebug symbiosis) metabolic model (i) reaction list and (ii) metabolite list. (b) Stand-alone Sodalis metabolic model (i) reaction list and (ii) metabolite list. (c) Stand-alone Sulcia (sharpshooter symbiosis) metabolic model (i) reaction list and (ii) metabolite list. (d) Stand-alone Baumannia metabolic model (i) reaction list and (ii) metabolite list. (e) Stand-alone Sulcia (cicada symbiosis) metabolic model (i) reaction list and (ii) metabolite list. (f) Stand-alone Hodgkinia metabolic model (i) reaction list and (ii) metabolite list. (g) Stand-alone spittlebug metabolic model (i) reaction list and (ii) metabolite list. (h) Stand-alone sharpshooter metabolic model (i) reaction list and (ii) metabolite list. (i) Stand-alone cicada metabolic model (part i) reaction list and (part ii) metabolite list. (j) Orphan reactions in spittlebug, sharpshooter, and cicada metabolic models. (k) Three-compartment Sulcia-Sodalis-spittlebug model (i) reaction list and (ii) metabolite list. (l) Three-compartment Sulcia-Baumannia-sharpshooter model (i) reaction list and (ii) metabolite list. (m) Three-compartment Sulcia-Hodgkinia-cicada model (i) reaction list and (ii) metabolite list. (n) Transcriptome constraints applied to insect compartment reactions. (o) Insect (bacteriocyte) media used for simulations. Download Table S5, XLSX file, 0.90 MB. Copyright © 2018 Ankrah et al. 2018 Ankrah et al. This content is distributed under the terms of the Creative Commons Attribution 4.0 International license.

The three-compartment model for each symbiosis (iNA761 [spittlebug], iNA629 [sharpshooter], and iNA533 [cicada] [Table S5k to m]) was reconstructed by integration of the models of each bacterial partner and their insect host, together with transport reactions to connect the three compartments (see Text S1 for details). Due to the dearth of annotated transporters in endosymbiont genomes and lack of empirical data on the energetic costs associated with metabolite transport between endosymbionts and their insect hosts, we adopted a parsimonious metabolite transport strategy in which the endosymbionts and insect hosts do not incur energetic costs for metabolite transfer. To set biologically relevant reaction fluxes, normalized gene expression data of the bacteriocyte were used to set lower and upper bounds for each host reaction (Table S5n). Missing host reactions, reactions with no matching transcript in the transcriptome assembly, were assigned arbitrary upper bounds of 10 mmol g dry weight^−1^ h^−1^ (with lower bounds of −10 mmol g dry weight^−1^ h^−1^ for reversible reactions). Approximately 66% of all host-constrained reactions carried flux under optimal conditions (Table S5n).

All model simulations applied aerobic conditions (maximum oxygen uptake flux of 20 mmol g dry weight^−1^ h^−1^) and a minimal external medium (insect hemolymph) comprising glucose, ammonia, and sulfate as carbon, nitrogen, and sulfur sources, respectively, universal metabolites present in the external medium of all three insect models, and nicotinate d-ribonucleotide (spittlebug model medium), fructose (sharpshooter model medium), and thiamine diphosphate, nicotinate d-ribonucleotide, dihydropteroate, pyridoxine 5-phosphate, pantothenate, and cobalt (cicada model medium). The maximum uptake flux for each reaction was capped at 100 mmol g dry weight^−1^ h^−1^. Amino acids were excluded as nutrient sources in all model simulations (Table S5o). In the absence of empirical data on the relative abundance of each symbiont within each insect host, we assumed equal biomass proportions for each symbiont in all our simulations by fixing the lower bound of the biomass reaction for each bacterium at 0.01 mmol g dry weight^−1^ h^−1^.

For the three-compartment model simulations, a single objective function representing the total amino acid content in the whole insect body and the insect B vitamin requirement was used. Amino acid coefficients were estimated from the total abundance of each amino acid in insect protein (see Table S6a to i in the supplemental material) following standard protocols (67, 68), and B vitamins were assigned arbitrary small coefficients (0.00005). The coefficients for biomass reaction components for individual bacterial models (Table S6a, d, and g) were derived from the biomass equation of metabolic model iSM199 of the insect symbiont Buchnera (12), modified to account for differences in the structural and biosynthetic needs of each symbiont. For example, Sulcia and Hodgkinia do not have a cell wall or the genetic capacity for cell wall synthesis, and consequently, cell wall components were omitted from their respective biomass equations. Amino acids and most central carbon intermediates were assigned the same biomass coefficients for all bacterial partners.

10.1128/mBio.01433-18.10 TABLE S6 (a**)** Objective function components in Sulcia, Sodalis, and spittlebug metabolic models. (b**)** Estimation of amino acid percentage in spittlebug protein. (c) Calculation of amino acid stoichiometry for spittlebug model. (d) Objective function components in Sulcia, Baumannia, and sharpshooter metabolic models. (e) Estimation of amino acid percentage in sharpshooter protein. (f) Calculation of amino acid stoichiometry for sharpshooter model. (g) Objective function components in Sulcia, Hodgkinia, and cicada metabolic models. (h) Estimation of amino acid percentage in cicada protein. (i) Calculation of amino acid stoichiometry for cicada model. Download Table S6, XLSX file, 0.08 MB. Copyright © 2018 Ankrah et al. 2018 Ankrah et al. This content is distributed under the terms of the Creative Commons Attribution 4.0 International license.

Metabolites exchanged between host and symbiont partners were identified by flux balance analysis (FBA) (69) and flux variability analysis (FVA) (28). With the exception of minerals and metabolites involved in cofactor biosynthesis which are required in small quantities by host and symbionts, a metabolite was considered to be imported/exported by a symbiont if the flux through its transport reaction was greater than 10^−6 ^mmol g dry weight^−1^ h^−1^.

### Calculation of symbiont maintenance costs.

For analyses of symbiont maintenance costs, flux through the biomass equation for a primary or coprimary symbiont was fixed to zero, while allowing flux through all other symbiont-associated reactions (so ensuring host access to essential nutrients), and the cost was computed as the difference between host growth yields in the presence and absence of symbiont biomass production. Applying these constraints allowed the costs associated exclusively with symbiont maintenance to be decoupled from the costs of meeting the EAA demands of the host. For all maintenance cost simulations, the uptake fluxes for the main sources of C, N, P, and S (glucose, fructose, ammonium, phosphate, and sulfate) were capped at the observed uptake fluxes in the three-compartment model (i.e., with both symbionts).

### Accession number(s).

The GenBank accession numbers of the sequences described here are NJPN00000000, NKXM00000000, MIEN00000000, NZ_NJPO00000000, and NJHQ00000000 for the bacterial genome sequences and PRJNA341855, PRJNA342845, and PRJNA343314 for the insect transcriptomes.

### Data availability.

All models have been provided in three formats—SBML (.xml), MATLAB (.mat), and Excel (.xls)—and deposited in GitHub (https://github.com/Bessem06/Hemipteran). SBML files of the models have also been submitted to the BioModels database (70) with the following identifiers: MODEL1806250003, MODEL1806250004, and MODEL1806250005.
