# Supplementary material for: The Cost of Metabolic Interactions in Symbioses between Insects and Bacteria with Reduced Genomes
Source: mBio. 2018 Sep 25;9(5):e01433-18. doi: 10.1128/mBio.01433-18 (PMC6156193; doi:10.1128/mBio.01433-18)
Supplement: FIG S1 [file mbo005184075sf1.pdf]

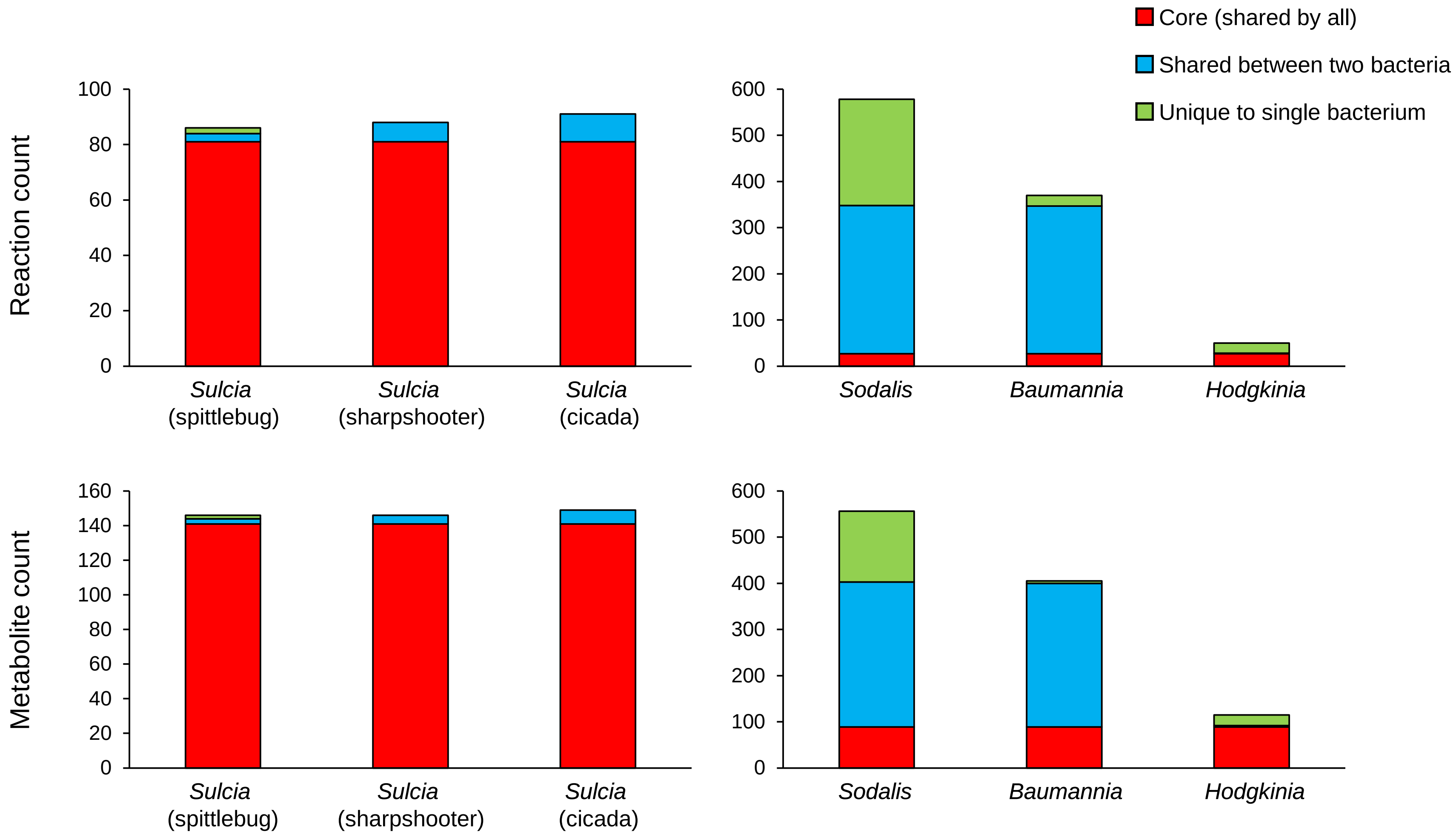

Figure S1. Overview of reactions and metabolites for xylem feeder bacterial symbionts. Reactions and metabolites are colored red (shared between all three primary or companion bacterial partners), blue (shared between any two primary or companion bacterial partners) and green (unique to a single bacterium).
