# Supplementary material for: The Cost of Metabolic Interactions in Symbioses between Insects and Bacteria with Reduced Genomes
Source: mBio. 2018 Sep 25;9(5):e01433-18. doi: 10.1128/mBio.01433-18 (PMC6156193; doi:10.1128/mBio.01433-18)
Supplement: FIG S2 [file mbo005184075sf2.pdf]

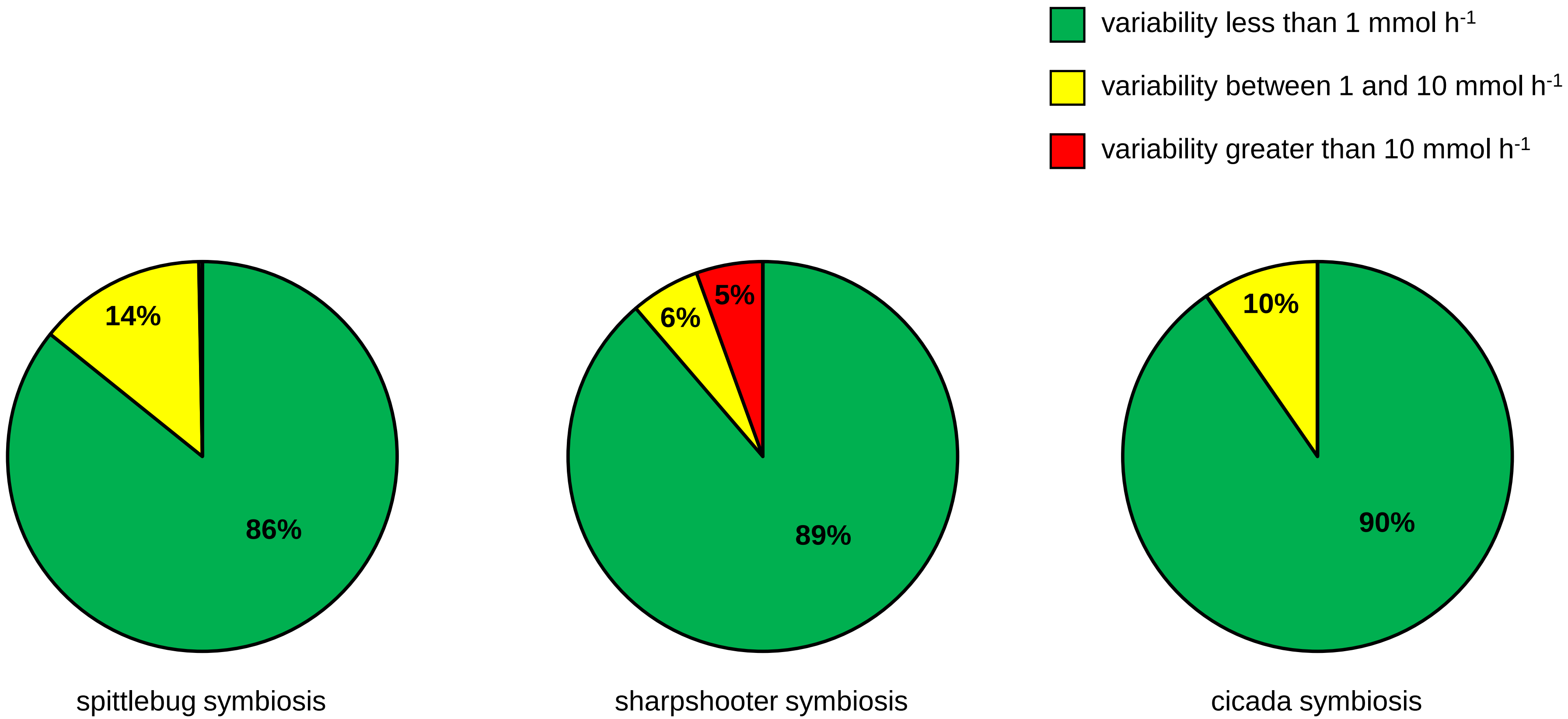

Figure S2. Variability in metabolic flux predictions in spittlebug, sharpshooter and cicada symbioses analyzed by flux variability analysis (FVA). Flux ranges are calculated as the difference between the maximum and minimum flux distributions resulting in the same growth objective value.
