# Supplementary material for: The Cost of Metabolic Interactions in Symbioses between Insects and Bacteria with Reduced Genomes
Source: mBio. 2018 Sep 25;9(5):e01433-18. doi: 10.1128/mBio.01433-18 (PMC6156193; doi:10.1128/mBio.01433-18)
Supplement: FIG S3 [file mbo005184075sf3.pdf]

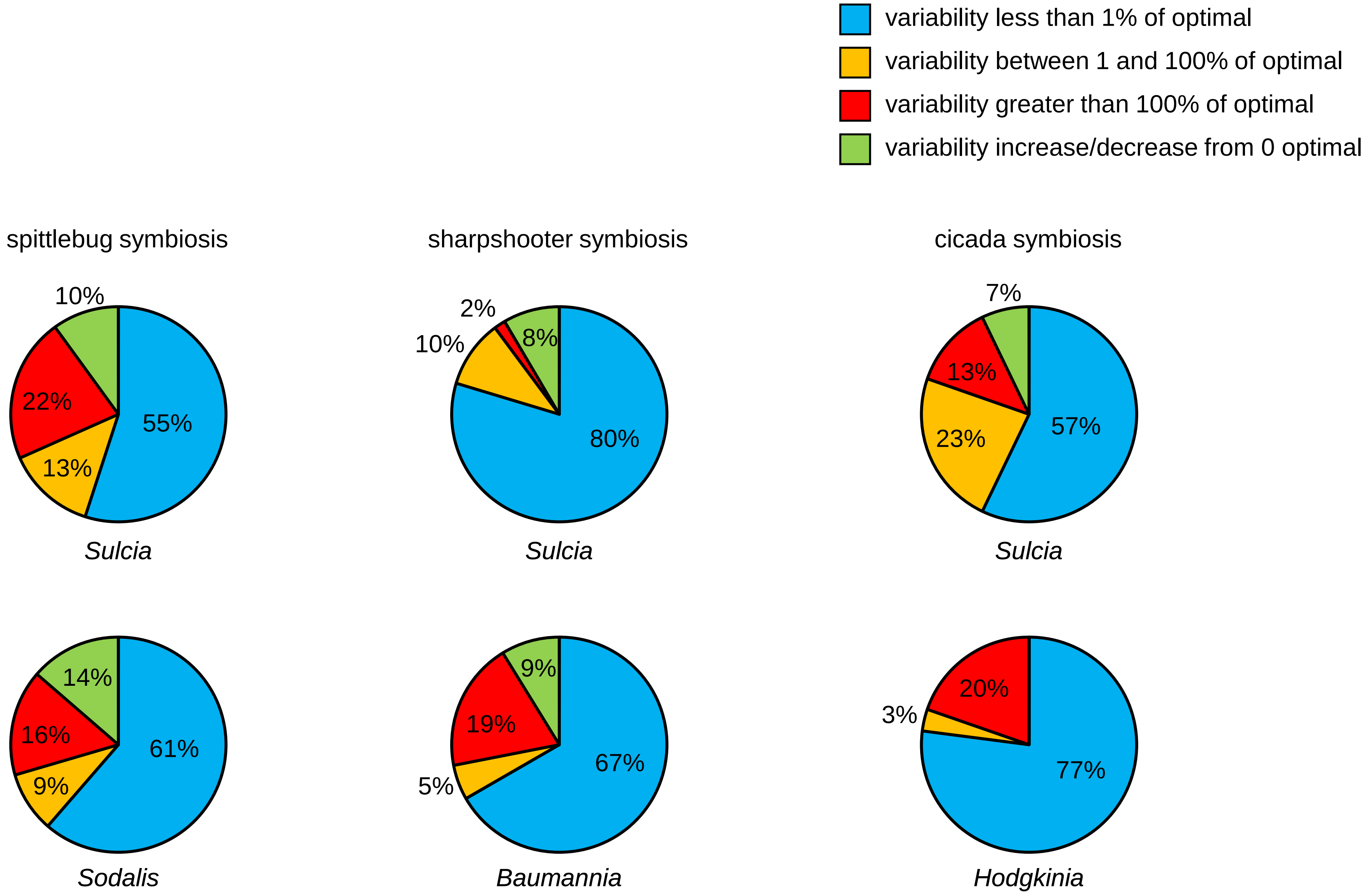

Figure S3. Variation in symbiont transport flux relative to optimal (maximum) flux in spittlebug, sharpshooter and cicada symbioses. Variation around maximum is calculated by diving the range of flux variation (obtained by FVA) by the optimal flux (calculated by FBA) and expressed as a percentage.
