# Supplementary material for: The Cost of Metabolic Interactions in Symbioses between Insects and Bacteria with Reduced Genomes
Source: mBio. 2018 Sep 25;9(5):e01433-18. doi: 10.1128/mBio.01433-18 (PMC6156193; doi:10.1128/mBio.01433-18)
Supplement: TEXT S1 [file mbo005184075s1.docx]

**Text S1**

**Supplementary Methods: Reconstruction and analysis of metabolic models**

**Metabolic model reconstruction**

The procedure of Ankrah et al. (1) was applied to reconstruct the genome-scale metabolic model for each of the six bacterial symbionts: *Sulcia* (*i*NA82) and *Sodalis* (*i*NA400) from the spittlebug, *Sulcia* (*i*NA74) and *Baumannia* (*i*NA234) from the sharpshooter, and *Sulcia* (*i*NA83) and *Hodgkinia* (*i*NA37) from the cicada (Table S5a–f). Briefly, reciprocal blasts of bacterial genomes against *Escherichia coli* str. K-12 substr. MG1655 were used to identify gene orthologs, which were compared to the *E. coli* str. K-12 substr. MG1655 metabolic model *i*JO1366 (2), and reactions encoded by these genes manually extracted to create a draft model. Organism-specific features and genes encoding metabolic reactions absent in *i*JO1366 were identified by literature review and searches of BioCyc, KEGG, EcoCyc, BiGG and BRENDA databases (3-7), and then added to the draft model.

To generate the transcriptome-derived metabolic models of the insects (Table S5g-i), we first identified reactions in each bacterial model that were predicted to consume or produce dead-end metabolites. Using homologous genes from *Drosophila* *melanogaster* and the pea aphid *Acyrthosiphon pisum*, the transcripts associated with each reaction were queried in the host transcriptome. The criteria for the bidirectional matches included in the model were: E value <1e-5, and >40% amino acid sequence identity. Identified reactions were incorporated into a draft reconstruction of the host metabolic network. Additional host reactions completing metabolic pathways for each added reaction were incorporated into a final reconstruction of the metabolic network. Orphan reactions (non-gene associated reactions) were then added to fill gaps in all the metabolic networks of all bacterial and insect models. Table S5j lists the orphan reactions. With the exception of the objective function, exchange and demand reactions, all reactions included in each model were mass and charge balanced. All metabolic networks were visualized using Cytoscape_v3.4.0 (8).

**Reconstruction of the integrated three-partner metabolic models**

To integrate the models of each bacterial partner and their insect host into a single three-compartment model, reactions and metabolites assigned to each partner were renamed, as follows: for the integrated spittlebug model, reactions in SUL (*Sulcia*), SOD (*Sodalis*), PHI (spittlebug), and metabolites in [s] (*Sulcia*), [d] (*Sodalis*), [p] (spittlebug); for the integrated sharpshooter model, reactions in SUL (*Sulcia*), BAU (*Baumannia*), GRA (sharpshooter), and metabolites in [s] (*Sulcia*), [b] (*Baumannia*), [g] (sharpshooter); and for the integrated cicada model, reactions in SUL (*Sulcia*), HODG (*Hodgkinia*), and NEO (cicada), and metabolites in [s] (*Sulcia*), [h] (*Hodgkinia*), and [n] (cicada). The stoichiometric matrices of each integrated symbiosis were combined to generate three-compartment integrated models *i*NA761 (spittlebug), *i*NA629 (sharpshooter) and *i*NA533 (cicada) (Table S5k-m). Transport reactions were added to connect the compartments of each bacterium to the host compartment, which was adopted as the site for metabolite exchange with the external environment.

Transport reactions for each bacterial compartment were selected by the following criteria within the metabolic models: 1) dead-end metabolites in each bacterial compartment were assigned transport reactions to or from the host compartment to allow enzymatic reactions mediating their synthesis or consumption to carry flux, and 2) metabolites produced in one species compartment for which another species compartment was auxotrophic were assigned transport reactions to and from the host compartment. Bacterial genome and host transcriptome data were not used to constrain transport reactions because annotated substrate specificities of transporters are notoriously unreliable (e.g. see (9)). The COBRA Toolbox version 3.0 ([10) run in Matlab (The MathWorks Inc., Natick, MA), was used for model testing and to determine metabolic flux distributions in each model using the Gurobi solver (11).

**Integration of gene expression data into metabolic models**

To set the host reaction bounds of the metabolic models, normalized host gene expression data (average TPM values) were applied as lower and upper bounds for each reaction (Table S5n), constraining our models to predict reaction fluxes of biologically relevant magnitudes. Normalized gene expression data were applied as both upper and lower bounds for reversible reactions, and as an upper boundary for each host reaction for non-reversible reactions (with the lower boundary maintained at zero) (Table S5n). Missing host reactions were assigned arbitrary upper bounds of 10 mmol gDW^-1^ h^-1^ (and -10 mmol gDW^-1^ h^-1^ lower bounds for reversible reactions). Table 5n reports the host-constrained reactions that carried flux under optimal conditions, accounting for 66% of the total.

**Metabolic model media composition and reaction constraints**

We customized the model medium used previously for a phloem-feeding insect symbiosis (1) for the xylem-feeding insects. Briefly, all model simulations applied aerobic conditions (maximum oxygen uptake rate of 20 mmol gDW^-1^ h^-1^), and a minimal external medium (insect hemolymph) comprising glucose, ammonia and sulfate as carbon, nitrogen and sulfur sources, respectively. Water, carbon dioxide, Iron (II), Iron (III) and phosphate were allowed to diffuse freely across all compartment membranes. In addition to the universal metabolites, nicotinate D-ribonucleotide was added to the spittlebug model medium, fructose to the sharpshooter model medium and thiamine diphosphate, nicotinate D-ribonucleotide, dihydropteroate, pyridoxine 5-phosphate, pantothenate and cobalt to the cicada model medium. Maximum uptake flux of each medium component was capped at 100 mmol gDW^-1^ h^-1^, with upper and lower bounds of all host compartment reactions constrained by transcript abundance of their associated genes. In the absence of empirical data on the relative abundance of the bacteria in each host, the lower bound of the biomass reactions of all bacteria were constrained to 0.01 mmol gDW^-1^ h^-1^.

**Objective function**

A single objective function representing the total amino acid content in the whole insect body and the insect B vitamin requirement was optimized for each three-compartment model simulation. Objective function amino acid coefficients were estimated from the total abundance of each amino acid in insect protein and quantified as previously described (1]) (Table s6A-i). Objective function B vitamins were assigned arbitrary small coefficients (0.00005) to ensure their production by each model. The coefficients for components of the biomass reaction for the individual bacterial models (Table S6A, D, G) were derived from the biomass equation of metabolic model *i*SM199 of the insect symbiont *Buchnera* (12), while taking into account differences in the structural and biosynthetic capabilities of each symbiont. For example, cell wall components were omitted from the biomass equations of *Sulcia* and *Hodgkinia* because these bacteria lack the genetic capability to synthesize cell walls.

**References**

1. Ankrah NYD, Luan J, Douglas AE. 2017. Cooperative metabolism in a three-partner insect-bacterial symbiosis revealed by metabolic modeling. J Bac 199: e00872-16. doi: 10.1128/JB.00872-16.

2. Orth JD, Conrad TM, Na J, Lerman JA, Nam H, Feist AM, Palsson BO. 2011. A comprehensive genome-scale reconstruction of Escherichia coli metabolism. Mol Sys Biol 7:535. doi: 10.1038/msb.2011.65.

3. Caspi R, Foerster H, Fulcher CA, Kaipa P, Krummenacker M, Latendresse M, Paley S, Rhee SY, Shearer AG, Tissier C*,* et al. 2008. The MetaCyc Database of metabolic pathways and enzymes and the BioCyc collection of Pathway/Genome Databases. Nucleic Acids Res. 36: D623-31. doi: 10.1093/nar/gkm900.

4. Kanehisa M, Goto S. 2000. KEGG: kyoto encyclopedia of genes and genomes. Nucleic Acids Res. 28: 27-30.

5. Keseler IM, Mackie A, Peralta-Gil M, Santos-Zavaleta A, Gama-Castro S, Bonavides-Martinez, C., Fulcher, C., Huerta, A.M., Kothari, A., Krummenacker, M. et al. 2013. EcoCyc: fusing model organism databases with systems biology. Nucleic Acids Res. 41: D605-12. doi: 10.1093/nar/gks1027.

6. Schellenberger J, Park JO, Conrad TM, Palsson BO. 2010. BiGG: a Biochemical Genetic and Genomic knowledgebase of large scale metabolic reconstructions. BMC Bioinformatics 11:213. doi: 10.1186/1471-2105-11-213. PubMed PMID: 20426874

7. Schomburg I, Chang A, Ebeling C, Gremse M, Heldt C, Huhn G, Schomburg, D.2004. BRENDA, the enzyme database: updates and major new developments. Nucleic Acids Res. 32: D431-3. doi: 10.1093/nar/gkh081.

8. Shannon P, A, Ozier O, Baliga NS, Wang JT, Ramage D, Amin N, Schwikowski B, Ideker, T. 2003. Cytoscape: a software environment for integrated models of biomolecular interaction networks. Genome Res 13:2498-2504. doi: 10.1101/gr.1239303.

9. Price DR, Feng H, Baker JD, Bavan S, Luetje CW, Wilson AC. 2014. Aphid amino acid transporter regulates glutamine supply to intracellular bacterial symbionts. Proc Natl Acad Sci U S A 111:320-325. doi: 10.1073/pnas.1306068111.

10. Schellenberger J, Que R, Fleming RM, Thiele I, Orth JD, Feist AM, Zielinski DC, Bordbar A, Lewis NE, Rahmanian S. 2011. Quantitative prediction of cellular metabolism with constraint-based models: the COBRA Toolbox v2.0. Nat Protoc 6:1290-1307. doi: 10.1038/nprot.2011.308.

11. Gurobi Optimization I (2016) Gurobi Optimizer Reference Manual. http://www.gurobi.com.

12. Macdonald SJ, Lin GG, Russell CW, Thomas GH, Douglas AE. 2012. The central role of the host cell in symbiotic nitrogen metabolism. Proc Biol Sci. 279:2965-73. doi: 10.1098/rspb.2012.0414.
